# Are Tuberculosis Patients in a Tertiary Care Hospital in Hyderabad, India Being Managed According to National Guidelines?

**DOI:** 10.1371/journal.pone.0030281

**Published:** 2012-01-17

**Authors:** Kiran Kumar Kondapaka, Surapaneni Venkateswara Prasad, Srinath Satyanarayana, Subhakar Kandi, Rony Zachariah, Anthony David Harries, Sharath Burugina Nagaraja, Shailaja Tetali, Raghupathy Anchala, Nanda Kishore Kannuri, Krishna Murthy, Dhanamurthy Koppu, Latha Vangari, Sreenivas Rao

**Affiliations:** 1 Department of Pulmonary Medicine, Osmania Medical College, Hyderabad, India; 2 International Union Against Tuberculosis and Lung Disease (The Union), South East Asia Regional Office, New Delhi, India; 3 Medecins sans Frontieres, Medical Department (Operational Research), Brussels Operational Center, Brussels, Luxembourg; 4 International Union Against Tuberculosis and Lung Disease (The Union), Paris, France; 5 Department of Infectious and Tropical Diseases, London School of Hygiene and Tropical Medicine, London, United Kingdom; 6 Office of the WHO Representative in India, New Delhi, India; 7 Public Health Foundation of India (Indian Institute of Public Health-Hyderabad), Hyderabad, India; 8 Department of Pulmonary Medicine, Gandhi Medical College, Hyderabad, India; 9 Department of Pediatrics, Osmania Medical College, Hyderabad, India; 10 State Tuberculosis Office, Andhra Pradesh, India; McGill University, Canada

## Abstract

**Setting:**

A tertiary health care facility (Government General and Chest hospital) in Hyderabad, India.

**Objectives:**

To assess a) the extent of compliance of specialists to standardized national (RNTCP) tuberculosis management guidelines and b) if patients on discharge from hospital were being appropriately linked up with peripheral health facilities for continuation of anti-Tuberculosis (TB) treatment.

**Methods:**

A descriptive study using routine programme data and involving all TB patients admitted to inpatient care from 1^st^ January to 30^th^ June, 2010.

**Results and Conclusions:**

There were a total of 3120 patients admitted of whom, 1218 (39%) required anti-TB treatment. Of these 1104 (98%) were treated with one of the RNTCP recommended regimens, while 28 (2%) were treated with non-RNTCP regimens. The latter included individually tailored MDR-TB treatment regimens for 19 patients and adhoc regimens for nine patients. A total of 957 (86%) patients were eventually discharged from the hospital of whom 921 (96%) had a referral form filled for continuing treatment at a peripheral health facility. Formal feedback from peripheral health facilities on continuation of TB treatment was received for 682 (74%) patients. In a tertiary health facility with specialists the great majority of TB patients are managed in line with national guidelines. However a number of short-comings were revealed and measures to rectify these are discussed.

## Introduction

India is one of the high tuberculosis (TB) burden countries in the world accounting for nearly 20% of the global incidence of 9.4 million TB cases [1]. The Government of India has been implementing a Revised National Tuberculosis Control Programme (RNTCP) since 1997 in order to control the national TB burden [2]. All health facilities including tertiary care centers are required to follow standardized RNTCP guidelines for the management of TB. In 2009, 92,071 (10%) smear positive cases notified under RNTCP were diagnosed in tertiary care hospitals including medical college hospitals [3].

The Government General and Chest Hospital (GG&CH) in Hyderabad, India is one of the main tertiary care centres in the state of Andhra Pradesh, where patients with TB requiring inpatient care are admitted. This specialized hospital receives patients from all over the state and beyond, and is well resourced with specialized chest physicians and sophisticated diagnostic facilities and has access to various types of anti-TB drugs. There is a concern that specialized clinicians in such hospitals might not be strictly following standardized RNTCP management guidelines as they might be inclined to tailor TB management including drug regimens to the specific needs of individual patients.

There is no published literature on whether such specialized centers comply with stipulated national TB management guidelines. In addition, no information exists on whether these patients successfully link up with peripheral health facilities for continuation of their anti-TB treatment after discharge from hospital inpatient care.

In this study we thus assessed a) the extent of compliance of the GGCH to RNTCP management guidelines and b) if patients on discharge were being appropriately linked up with peripheral health facilities for continuation of anti-TB treatment.

## Methods

### Design

This was a descriptive study involving a retrospective record review.

### Study setting and population

GG&CH is a 670 bed hospital, linked to two medical colleges providing out-patient and in-patient care for TB and chest diseases. All patients presenting to the out patient department of the hospital are examined and those diagnosed with all TB and meriting in-patient admission receive anti-TB treatment on the wards. The decision to admit the TB patients for inpatient care rests with the specialist physicians depending on their assessment of the clinical seriousness of the patients and there are no uniform standard clinical or other criteria for admission. Once their condition becomes stable enough to allow discharge, they continue anti-TB treatment in a peripheral health facility.

The RNTCP follows WHO recommended guidelines for the management of TB patients [2], [4]. All TB patients requiring anti-TB treatment are categorized and treated with thrice weekly intermittent anti- TB treatment in a supervised manner. (**Table 1**)

**Table 1:** Treatment categories and regimens for TB patients in India.

Treatment category	Type of patients	Treatment regimens***	
		Intensive Phase	Continuation phase
Category 1	New sputum smear-positive PTB New sputum smear-negative PTB, seriously ill* New EPTB, seriously ill*	2(H_3_R_3_Z_3_E_3_)	4(H_3_R_3_)
Category 2	Sputum smear-positive relapse Sputum smear-positive treatment failure Sputum smear-positive treatment after default	2(H_3_R_3_Z_3_E_3_S_3_)+1(H_3_R_3_Z_3_E_3_)	5(H_3_R_3_E_3_)
Category 3	New sputum smear-negative, not seriously ill** New EPTB, not seriously ill**	2(H_3_R_3_Z_3_)	4(H_3_R_3_)
Category 4	All patients with diagnosed Multidrug resistant TB	6 (9) Km levo Eto Cs Z E	18 Ofx Eto Cs E

PTB = Pulmonary tuberculosis: EPTB = Extra pulmonary tuberculosis.

*In children, seriously ill sputum smear-negative PTB includes all forms of sputum smear-negative PTB other than primary complex. Seriously ill EPTB includes TB meningitis (TBM), disseminated TB, TB pericarditis, TB peritonitis and intestinal TB, bilateral extensive pleurisy, spinal TB with or without neurological complications, genitourinary TB, and bone and joint TB.

**Not seriously ill sputum smear-negative PTB includes primary complex. Not seriously ill EPTB includes lymph node TB and unilateral pleural effusion.

***Prefix indicates month and subscript indicates thrice weekly.

H = Isoniazid, R = Rifampicin, Z = Pyrazinamide, E = Ethambutol, S = Streptomycin, Km = Kanamycin, Levo = Levofloxacin, Eto = Ethionamide, Cs = Cycloserine,

During the hospital stay, all patients are registered and a treatment card is provided. Directly observed treatment (DOTS) is provided by the ward staff.

On discharge from the hospital ward, patients are formally referred (by filling a referral for treatment form) to a DOTS TB unit (TU) closest to the patient's residence for continuation of anti-TB treatment. The Medical officer of the respective unit sends a formal feedback (usually by post) to the hospital acknowledging reception of the patient at the peripheral facility by filling a feedback form which will reach the tertiary care facility by post or in person from the respective district TB officer. All referrals and feedbacks are documented on a ‘referral for treatment’ register maintained at GG&CH and updated on a daily basis.

The study population included all patients admitted to the inpatient ward with a diagnosis of TB during the period 1^st^ January to 30^th^ June 2010.

### Assessing compliance to RNTCP management guidelines

Data collected from case records and treatment cards of those admitted to the GG&CH wards with a diagnosis of TB were assessed to measure compliance to one of the recommended RNTCP treatment regimens. If a patient was placed on an anti-TB regimen as recommended by RNTCP guidelines, this was defined as being compliant. If this was altered in any way, the status was designated as being non compliant with RNTCP guidelines. A successful linkage to continuing anti-TB care for a given patient was defined as physical availability of a feedback form, acknowledging reception of the referred patient at the peripheral health facility.

### Data collection and analysis

Data from the patient case records, the referral register and feedback forms were used to gather information related to this study. These data were entered into a pre-structured format on Microsoft Excel. The data were analyzed using Epi-Info (version 6.0 CDC, Atlanta, USA).

### Ethics Approval

The study was approved by the Ethics Advisory Group of the International Union against Tuberculosis and Lung Diseases, the Institutional Ethics Committee of the Public Health Foundation of India and by the institutional ethical committee of Government General and Chest Hospital. The activity was determined to be a retrospective programme evaluation of the implementation of national guidelines, hence individual patient consent was deemed unnecessary. Electronic databases created for this analysis were stripped of personal health identifiers and maintained securely.

## Results

### Characteristics of the study population

**Figure 1:**
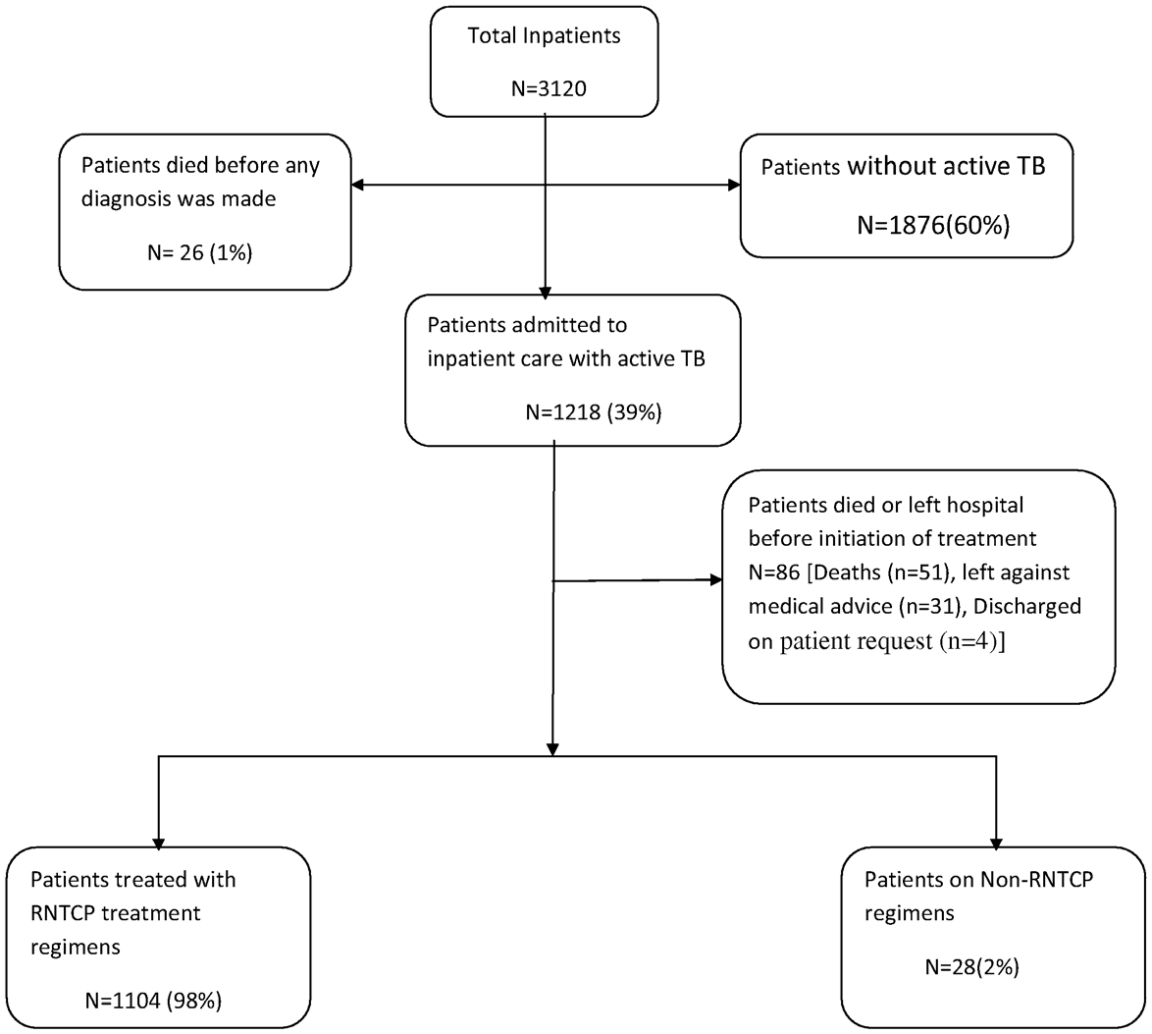
Type of tuberculosis treatment for tuberculosis patients admitted to the Government General and Chest Hospital, Hyderabad, India.

A total of 3120 individuals were admitted to GG&CH, of whom 1876 (60%) did not have active TB and 26 (1%) died before any diagnosis could be made. A total of 1218 patients were admitted to inpatient care with active TB and were thus included in the analysis **(** **Figure 1** **)**. These patients included 905 (74%) males, 1133 with pulmonary TB and 85 extra pulmonary TB cases.

### Compliance to existing RNTCP guidelines

Of 1218 patients with active TB, 86(7%) died or left hospital before initiating anti-TB treatment. A total of 1104(98%) were placed on a regimen that was included in RNTCP guidelines, 28 (2%) were placed on a non stipulated regimen. **(** **Figure 1** **)**. Of these 28 patients, 19 were placed on tailored MDR-TB treatment regimens based on drug sensitivity testing. The remaining nine patients were found to be on ad-hoc regimens started prior to admission at GG&CH but these regimens remained unchanged. **Table 2** shows the type of TB in relation to treatment regimens used. Despite being on an existing RNTCP regimen, 17 patients were placed on a non recommended RNTCP drug regimen category for reasons that were not specified in the patient records **(** **Table 2** **)**.

**Table 2:** Type of Tuberculosis and treatment regimens of TB patients admitted to Government General and Chest Hospital, Hyderabad, India (2010).

	Treatment regimens							
Type of tuberculosis		RNTCP regimens*					Non-RNTCP regimens	
	Total	Cat-1	Cat-2	Cat-3	Cat-4	Unknown regimens	MDR-TB regimens**	Ad-hoc regimens
New Tuberculosis cases	696	634	14	28	0	11	0	9
Retreatment cases	385	12	373	0	0	0	0	0
MDR-TB	51	0	0	0	32	0	19	0
Total	1132	646	387	28	32	11	19	9

*RNTCP- Revised National Tuberculosis Control Programme.

**Regimen formulated based on the drug susceptibility of the individual patient to first and second line anti- TB treatment.

Cat-1 = Category 1, Cat-2 = category 2, Cat-3 = Category 3, Cat-4 = Category 4.

### Successful linkage after discharge for continuing anti-TB treatment

**Figure 2:**
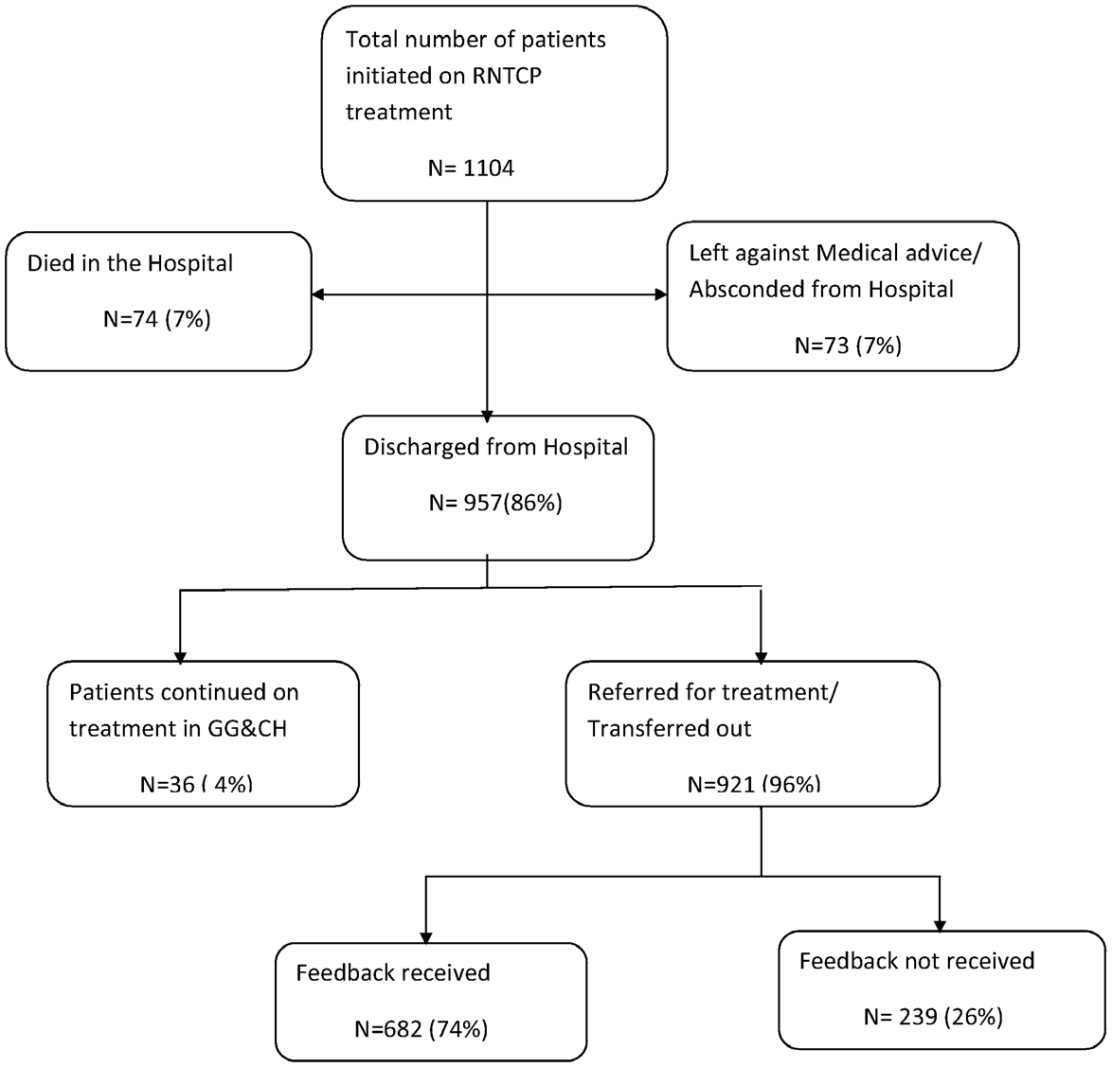
Referral for treatment and feedback status of patients initiated on RNTCP treatment Regimen at Government General and Chest Hospital, Hyderabad, India.

Out of the 1104 patients on a RNTCP regimen, 921(96%) were eventually referred for continuation of treatment at peripheral centres from where a formal feedback was received for 682 (74%) patients **(** **Figure 2** **)**.

## Discussion

This study shows that 98% of patients with active TB admitted to a tertiary facility in India are placed on an anti-TB drug regimen that is included in the RNTCP guidelines. Formal feedback on referral was received from peripheral faculties for seven out of ten discharged patents. This is the only published study in the literature assessing the degree of compliance of a tertiary facility with national guidelines and the findings are encouraging. Importantly, the study shows that the great majority of clinicians in such specialized facilities do indeed comply with the recommended national guidelines for TB management despite the concern that the contrary might be the case. However this study reveals a number of programmatic issues that merit consideration.

First, 19 patients were found to be on an “individually tailored” MDR-TB treatment. These patents belonged to districts that did not have access to a TB facility offering MDR-TB treatment (a so called DOTS-Plus facility). These patients had drug sensitivity testing performed and their drug regimens were then tailored according to the drug sensitivity patterns. Furthermore, the state Government covers the cost of treatment for these patients. Thus, the divergence from stipulated RNTCP guidelines is justified but this reflects the relative lack of access to DOTS-Plus sites in some districts in Andhra Pradesh. This situation needs to be improved. The lack of access to effective and standardized MDR-TB regimens tends to increase the dependence on tailored drug regimens [5], [6].

Second, nine patients who were started on an ad-hoc non RNTCP regimen before arriving at the GG&CH were simply continued and discharged on their non-recommended regimens. It would have been expected that these regimens be corrected by the tertiary facility.

Third, 17 patients were offered a drug regimen that was not recommended for their TB type. The reasons for this are unclear but this merits further investigation as it indicates non-compliance with national guidelines.

Finally, feedback on referrals was not received in three out of ten individuals referred to peripheral faculties for continuation of anti-TB treatment. These patients were lost to follow-up, indicating deficiencies in referral for treatment and feedback and such patients due to ineffective mechanisms may become defaulters or MDR patients. This highlights the need for better links and discussions with peripheral facilities as well as active tracing of feedback. Use of mobile telephones can be considered as a way to improve this linkage [7].

The strengths of this study are that a large number of TB patients were included in the study; information was gathered on an individual basis and cross-checked using patients records and cards and thus we believe that the data are robust and reliable. Since we used routine programme data, the findings are likely to reflect the operational reality on the ground. We also followed STROBE guidelines on reporting of observational studies [8]. This study faces the usual limitations of observational studies.

In conclusion, in a tertiary health facility in Hyderabad, India the great majority of TB patients are managed in line with national guidelines. A number of important short-comings were revealed and these need to be addressed.
